# Bronchoscopic closure of intubation-related bronchopleural fistulas using combined argon plasma coagulation and fibrin glue: a case series

**DOI:** 10.1186/s12890-026-04147-9

**Published:** 2026-02-04

**Authors:** Siavash Kooranifar, Ata jafrasteh, Taghi Riahi, Vahan Moradians, Banafshe Darvishi Teli, Tayeb Ramim

**Affiliations:** 1 Department of Internal Medicine, School of Medicine, Rasool Akram Medical Complex, Iran University of Medical Sciences, Tehran, Iran; 2 Health Management and Economics Research Center, Health Management Research Institute, Iran University of Medical Sciences, Tehran, Iran; 3 Student’s Scientific Research Center (SSRC), Tehran University of Medical Sciences, Tehran, Iran

**Keywords:** Bronchopleural fistula, Fibrin glue, Argon plasma coagulation, Endotracheal intubation, Bronchoscopy

## Abstract

**Background:**

Bronchopleural fistula (BPF) is a rare but potentially life-threatening complication, particularly following endotracheal intubation and prolonged mechanical ventilation. Delayed diagnosis or inadequate management may result in persistent air leakage, recurrent infection, prolonged hospitalization, and high mortality. Although surgical repair remains the standard treatment, it is often associated with considerable morbidity, especially in critically ill or high-risk patients. Consequently, minimally invasive bronchoscopic techniques have emerged as attractive alternatives.

**Methods:**

This case series evaluated the efficacy and safety of a combined bronchoscopic approach using argon plasma coagulation (APC) followed by fibrin glue instillation to close intubation-related bronchopleural fistulas. Six adult patients treated at Rasoul Akram Hospital, Tehran, Iran, during the 2024–2025 calendar year were included. All patients underwent the same intervention without a control group. APC was applied to de-epithelialize the fistula tract, followed by fibrin glue injection under bronchoscopic guidance. The primary outcome was complete fistula closure within 7 days. Secondary outcomes included maintenance of closure at 3 months, recurrence rate, and procedure-related complications.

**Results:**

Complete fistula closure was achieved in five of six patients (83.3%), with cessation of air leakage documented within 24–72 h in most cases and sustained throughout the 3-month follow-up period. One 78-year-old patient with severe comorbidities, including diabetes mellitus, chronic kidney disease, respiratory failure, and sepsis, died two days after the procedure; death was attributed to underlying systemic illness rather than the intervention. No procedure-related complications or fistula recurrence were observed among successfully treated patients.

**Conclusion:**

Combined APC and fibrin glue therapy appears to be a safe, cost-effective, and efficacious minimally invasive option for closing bronchopleural fistulas of varying sizes (small, medium, and selected large fistulas), particularly in patients who are poor surgical candidates. Careful patient selection and optimization of underlying conditions remain essential to achieving optimal outcomes.

**Supplementary Information:**

The online version contains supplementary material available at 10.1186/s12890-026-04147-9.

## Introduction

 Bronchopleural fistula (BPF) is an abnormal communication between the bronchial tree and the pleural space, representing a serious and potentially fatal complication if not managed adequately. Its most severe sequelae include persistent air leak, recurrent infections, refractory pneumothorax, prolonged hospitalization, and high mortality rates [1, 2]. While the reported incidence of BPF following major thoracic surgery is approximately 1.9% with mortality ranging from 18% to 50% depending on fistula severity and patient comorbidities [3, 4] this complication is increasingly encountered as a consequence of endotracheal intubation and prolonged mechanical ventilation in critical care settings [4].

While major pulmonary surgeries such as pneumonectomy and lobectomy remain the most common etiologies, the incidence of BPF related to endotracheal intubation and prolonged mechanical ventilation has increased in recent years [4, 5]. Mechanical ventilation can induce ischemic and pressure-related injury to the tracheobronchial wall, particularly through excessive cuff pressure, resulting in tissue necrosis and fistula formation [6]. This risk is heightened in patients with septic shock, acute respiratory distress syndrome (ARDS), or pre-existing tracheobronchial pathology [7, 8].

The pathophysiology of intubation-related BPF typically involves ischemic injury, pressure necrosis, secondary infection, and progressive disruption of airway integrity, ultimately leading to pathological communication with the pleural space [5, 9–11]. When diagnosis is delayed, these lesions may rapidly progress to uncontrolled air leakage, empyema, and death [12, 13].

Conventional management strategies for BPF include surgical repair, flap transposition, and omentoplasty. However, these procedures are frequently contraindicated in patients with poor general condition and are associated with high morbidity and mortality [14, 15]. Consequently, bronchoscopic techniques such as fibrin glue (FG) application and argon plasma coagulation (APC) have gained increasing attention as less invasive alternatives [3, 16].

Fibrin glue promotes local hemostasis and tissue repair by forming a fibrin matrix at the fistula site, while APC induces superficial coagulative necrosis and stimulates granulation tissue formation along the fistula tract [3, 17]. Although several case reports have demonstrated the effectiveness of fibrin glue alone or in combination with adjunctive techniques, robust clinical data on the combined use of APC and fibrin glue—particularly in intubation-related BPF remain limited [18–20].

Given the significant clinical burden of BPF and the need for effective minimally invasive therapies, this case series aimed to evaluate the efficacy and safety of combined APC and fibrin glue therapy for bronchopleural fistula closure in a selected patient population.

## Methods

### Study design and setting

This retrospective observational case series was conducted at Rasoul Akram Hospital, Tehran, Iran, during 2024–2025. Ethical approval was obtained from the institutional review board.

### Patient selection

Adult patients (≥ 18 years) with bronchoscopically confirmed BPF secondary to prolonged intubation were included. Diagnosis was supported by clinical symptoms, chest CT imaging, and bronchoscopic visualization. Comorbid conditions such as chronic obstructive pulmonary disease (COPD), diabetes, and renal or cardiac dysfunction were recorded.

### Intervention

Flexible bronchoscopy was performed under conscious sedation. APC at 40–60 W was applied to the fistula tract to induce controlled superficial coagulation and facilitate granulation. Immediately afterward, fibrin glue was instilled via a catheter under bronchoscopic guidance.

### Outcomes and follow-up

The primary outcome was complete fistula closure within 7 days, defined by the absence of air leak on clinical assessment and bronchoscopic confirmation. Secondary outcomes included sustained closure at three months, recurrence, and procedure-related complications.

## Results

Six patients (five males and one female) with a mean age of 57 ± 19.05 years were included. Fistula diameters ranged from less than 5 mm to greater than 8 mm.

Complete closure of the fistula was achieved in five patients (83.3%) within 7 days. In these cases, cessation of air leak occurred within 24 to 72 h and remained stable at the three-month follow-up.

One elderly patient with multiple comorbidities, including diabetes, chronic obstructive pulmonary disease (COPD), and renal impairment, was hospitalized due to sepsis and subsequently died following the intervention. Based on clinical evaluation, this event was not directly attributed to the bronchoscopic procedure.

No complications directly related to the intervention were observed during the follow-up period. Patient clinical characteristics and treatment outcomes are summarized in Table 1.

**Table 1:** Patients Characteristics and Outcomes of Bronchopleural Fistula Closure

Patient No.	Age (years)	Sex	Major Comorbidities	Fistula Size Category	Fistula Size (mm)	Initial Closure (≤ 24 h)	Complete Closure at 7 Days	Outcome at 3-Month Follow-up	Procedure-related Complications	Final Outcome
1	54	Male	COPD, diabetes mellitus, chronic kidney disease, sepsis	Medium	5–8	Yes	Yes	Sustained closure	None	Successful
2	27	Male	History of treated tuberculosis	Large	> 8	Yes	Yes	Sustained closure	None	Successful
3	45	Female	None	Medium	5–8	Yes	Yes	Sustained closure	None	Successful
4	78	Male	Open-heart surgery	*Two fistulas	< 5 and 5–8	No	No	Death (sepsis-related)	None	Failed
5	65	Male	COPD, diabetes mellitus	Medium	5–8	Yes	Yes	Sustained closure	None	Successful
6	73	Male	CHF, COPD	Large	> 8	Yes	Yes	Sustained closure	None	Successful

*Patient 4 had two separate fistulas (one small, one medium)

**Fig. 1:**
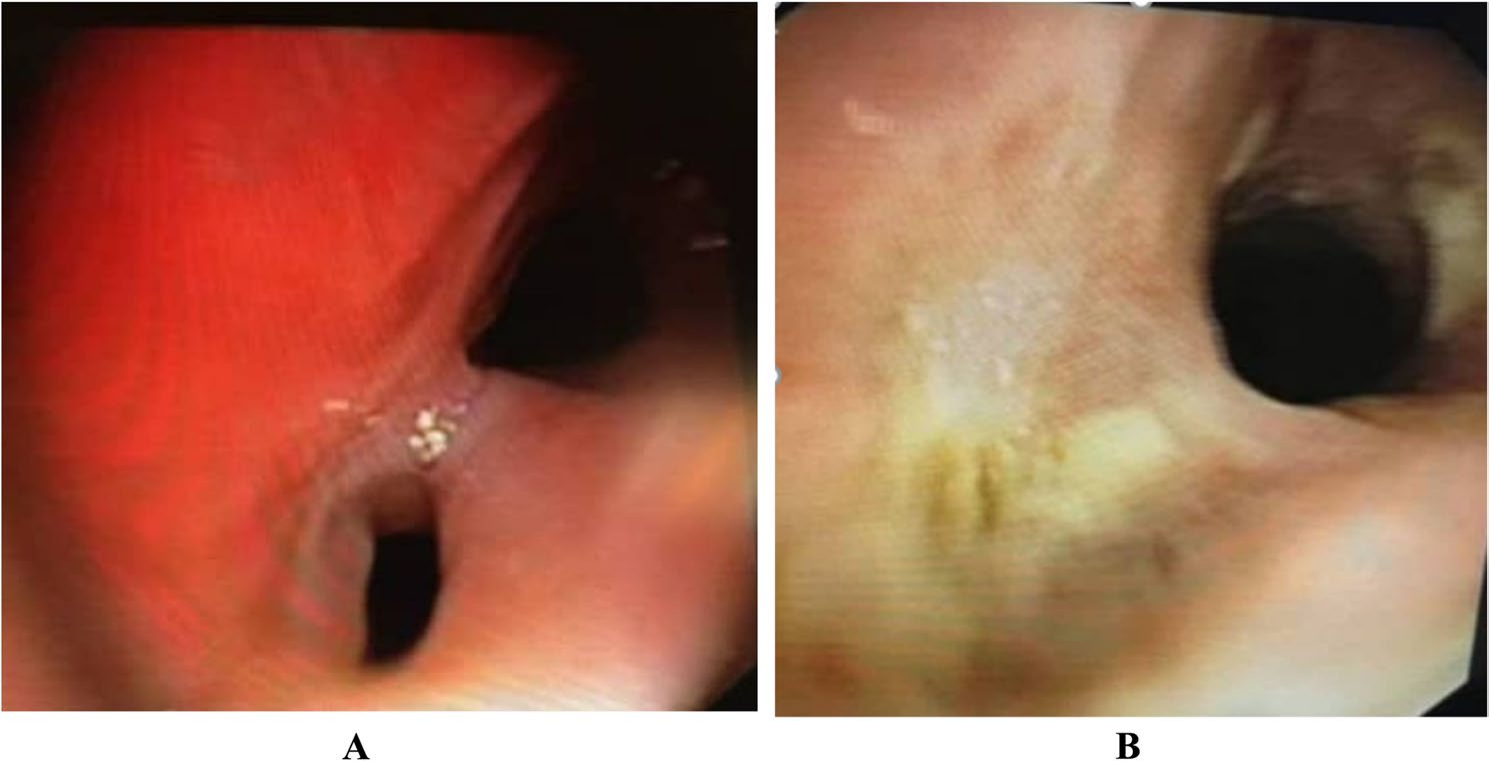
Endoscopic view of the carina in a patient with a large post-intubation bronchopleural fistula (BPF). **A** Before intervention: visible fistula tract with active air leak. The orifice of the BPF (arrow) is seen at the site of the right main bronchus, mimicking a closed bronchus due to tissue disruption and edema. Active air leak was observed from this opening. **B** After combined argon plasma coagulation (APC) and fibrin glue therapy: complete closure of the fistula with cessation of air leak. Complete closure of the fistula orifice is achieved. The underlying right main bronchus remained patent, and air leak ceased. No pneumonectomy (surgical or functional) was performed in any patient in this series

To demonstrate the treatment outcomes, Fig. 1 presents the endoscopic appearance of the carina in a patient with a large post-intubation bronchopleural fistula (BPF).

(A) Before intervention: The fistula orifice (indicated by the arrow) is visible at the origin of the right main bronchus, resembling a completely obstructed bronchus due to tissue disruption and edema. An active air leak was clearly observed at this site.
(B) After combined APC and fibrin glue therapy: The fistula opening shows complete closure following treatment. The right main bronchus remained patent, and the air leak fully resolved. Notably, none of the patients in this series required pneumonectomy, either surgical or functional.

## Discussion

### Overview of findings

In this retrospective observational case series of six patients with intubation-related bronchopleural fistulas, combined fibrin glue and argon plasma coagulation (APC) therapy achieved complete closure in five patients (83.3%) within seven days. Air leak cessation occurred within 24–72 h and remained stable at the three-month follow-up. No procedure-related complications were observed.

One elderly patient with multiple comorbidities, including diabetes, chronic obstructive pulmonary disease (COPD), and chronic kidney disease, was hospitalized due to sepsis and died following the intervention. Clinical evaluation suggested that the death was not directly related to the bronchoscopic procedure. This underscores the importance of cautious patient selection, particularly in elderly individuals with multiple comorbidities.

### Comparison with previous literature

Our findings are in line with previous reports on endoscopic management of bronchopleural fistulas. Takanami (2003) successfully treated two patients using a collagen patch coated with fibrin glue; however, closure took 9 weeks to 3 months [21]. In contrast, closure in most of our patients occurred within 24 h, suggesting a potential benefit from the addition of APC prior to fibrin glue. Unlike Takanami [21], who employed monotherapy, our sequential dual-modality approach may contribute to more rapid healing.

Ishikawa et al. (2013) reported a 75% success rate in four patients using combined collagen patch and fibrin glue, emphasizing careful patient selection [19]. Fiorelli et al. (2015) also reported 75% success using cellulose patches with submucosal fibrin glue [18]. Our observed 83.3% success rate is consistent with these studies, without overstating efficacy, and highlights that dual-modality therapy may offer additional advantages in selected patients.

### Mechanistic considerations

- Fibrin Glue: Provides a mechanical seal against air leakage and acts as a biological scaffold facilitating organized tissue repair and fibroblast migration [21].
- Argon Plasma Coagulation: Induces superficial coagulation and a localized inflammatory response, promoting granulation tissue formation and neovascularization, thus preparing the tissue bed for optimal fibrin glue adhesion [22].
- Synergistic Mechanism: The combination likely contributes to faster closure times observed in our series, compared with monotherapy approaches reported previously .

### Predictive factors and study limitations

Several factors may influence procedural outcomes:

1. Age: Advanced age may reduce tissue repair capacity and increase susceptibility to infection [23]. The single failure occurred in a 78-year-old patient, supporting careful consideration of age in patient selection.
2. Comorbidities: Conditions such as COPD, diabetes, and renal impairment can independently and collectively compromise tissue healing and immune function.
3. Fistula Size: Medium-sized fistulas (5–8 mm) responded favorably, aligning with previous reports [19]. Larger fistulas may require repeated sessions or may be less responsive.
4. Tissue Quality and Infection: Active infection or empyema can hinder closure, sometimes necessitating drainage or local debridement before endoscopic intervention [18, 21] .

The study’s sample size (*n* = 6) represents a limitation. However, the rarity of intubation-related bronchopleural fistulas and the limited patient population available during the study period justified inclusion of all eligible cases. While results may not be fully generalizable, they provide meaningful insight into the safety and feasibility of combined APC and fibrin glue therapy.

### Clinical implications

Compared with previous methods:

- Takanami’s Collagen-Fibrin Patch: Our patients achieved closure within 24 h versus 9 weeks–3 months; no repeat procedures were required [21].
- Open Surgical Repair: Endoscopic therapy avoids thoracotomy-related complications (20–50%), reduces recovery time, and maintains comparable or lower mortality [21].
- Bronchial Stents: APC and fibrin glue are more cost-effective, technically simpler, and do not require removal, offering practical advantages in high-risk patients.

### Novel contribution

This is the first reported series combining APC with fibrin glue for intubation-related bronchopleural fistulas. Sequential application appears to optimize tissue preparation, enhance adhesion, and accelerate closure, demonstrating a feasible and safe approach in selected high-risk patients. The procedure provides a minimally invasive, rapid, and cost-effective alternative to surgery or stenting, with sustained closure over three months.

### Limitations

This study has several important limitations that should be considered when interpreting the findings. First, the sample size of six patients is small. This limitation reflects both the rarity of intubation-related bronchopleural fistulas and the limited number of patients presenting to the study hospital during the enrollment period. Consequently, all eligible patients were included in the study, which, while necessary, restricts statistical power and limits the generalizability of the results.

Second, the study did not include a control group, such as patients treated with fibrin glue alone or alternative standard therapies. This prevents direct comparison and reduces the ability to draw definitive conclusions about the relative efficacy of the combined APC and fibrin glue approach.

Third, the follow-up period of three months, although sufficient for short-term assessment, may not capture late complications or delayed recurrences, highlighting the need for longer-term surveillance in future studies.

Finally, heterogeneity in patient characteristics—including differences in age, underlying comorbidities, and fistula size—may have influenced individual responses to treatment, introducing variability that limits the precision of outcome interpretation. Despite these limitations, the study provides meaningful insights into the feasibility and safety of combined APC and fibrin glue therapy in this rare clinical scenario.

## Conclusion

Combined argon plasma coagulation (APC) and fibrin glue therapy appears to be a feasible, safe, and effective bronchoscopic approach for the management of intubation-related bronchopleural fistulas. In this case series, the method achieved high closure rates with no procedure-related complications, demonstrating its potential as a minimally invasive alternative to conventional surgical or stent-based interventions. However, given the small sample size and the observational nature of the study, these findings should be interpreted cautiously. Further controlled studies involving larger patient cohorts and longer follow-up periods are warranted to validate efficacy, define optimal patient selection criteria, and assess long-term outcomes. 

## Supplementary Information

Supplementary Material 1.

Supplementary Material 2.

Supplementary Material 3.

Supplementary Material 4.

## Data Availability

The datasets used and/or analyzed during the current study are available from the corresponding author on reasonable request.

## Abbreviations

BPF: Bronchopleural fistula
APC: Argon Plasma Coagulation
FG: Fibrin Glue
ARDS: Acute Respiratory Distress Syndrome
COPD: Chronic Obstructive Pulmonary Disease
CHF: Congestive Heart Failure
