# Supplementary figures and images for: Bronchoscopic closure of intubation-related bronchopleural fistulas using combined argon plasma coagulation and fibrin glue: a case series
Source: BMC Pulm Med. 2026 Feb 4;26:103. doi: 10.1186/s12890-026-04147-9 (PMC12964874; doi:10.1186/s12890-026-04147-9)

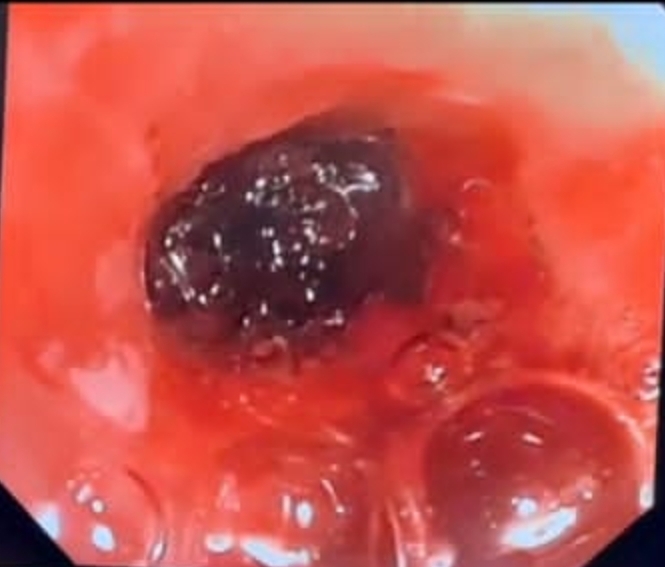

Supplement: Supplementary file 1 — Supplementary Material 1. [file 12890_2026_4147_MOESM1_ESM.jpg]
